# Supplementary material for: Assessing the stability of Pd-exchanged sites in zeolites with the aid of a high throughput quantum chemistry workflow
Source: Nat Commun. 2022 May 25;13:2910. doi: 10.1038/s41467-022-29505-z (PMC9133006; doi:10.1038/s41467-022-29505-z)
Supplement: Supplementary file 1 — Supplementary Information [file 41467_2022_29505_MOESM1_ESM.pdf]

Supplementary Information

Assessing the stability of Pd-exchanged sites in zeolites with the aid of a high throughput chemistry workflow

H. Aljama et al.

Supplementary Notes

Raw data is available in an open access data repository as specified in the Data Availability statement in the main paper. The nomenclature used to tabulate the data is as follows: zeolite name (CHA or BEA) - a number representing structure with a unique Al arrangement - adsorbate name (Pd^{+2}, $Pd^{+}H^{+}$, Pd^{+}, H^{+} or $H^{+}H^{+}$) - a number representing an initial position of the adsorbate - level of theory (GGA or hGGA) - type of calculations (opt for optimization and sp for single point calculation).

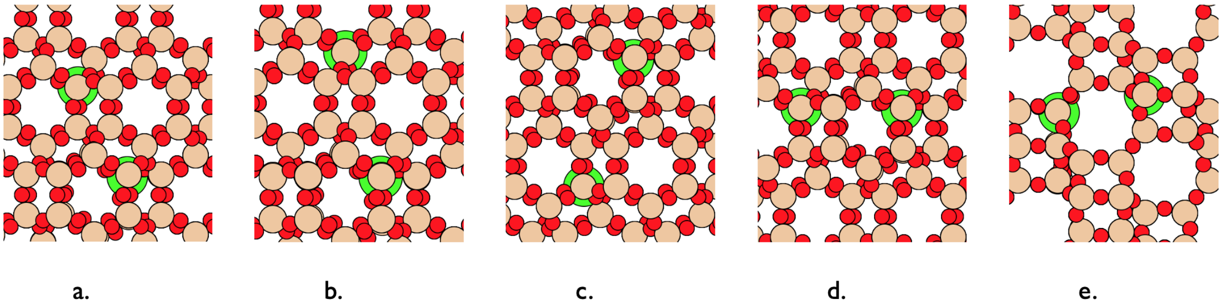

Supplementary Figure 1: Images of the optimized framework geometry of 5 different Pd^{+2}-exchanged-CHA structures where the Al atoms comprising the pair are in a 5N configuration (separated by 3 Si atoms). Color coding: red=oxygen, beige=silicon; the Al atoms are enlarged and are shown in green. The calculated formation energies of the structures are a. -0.72 b. -0.72 c. -0.84 d. -1.05 and e. -1.22 eV, which are relatively weaker than NNNN and NNN pairs, as shown in Supplementary Figure 2.

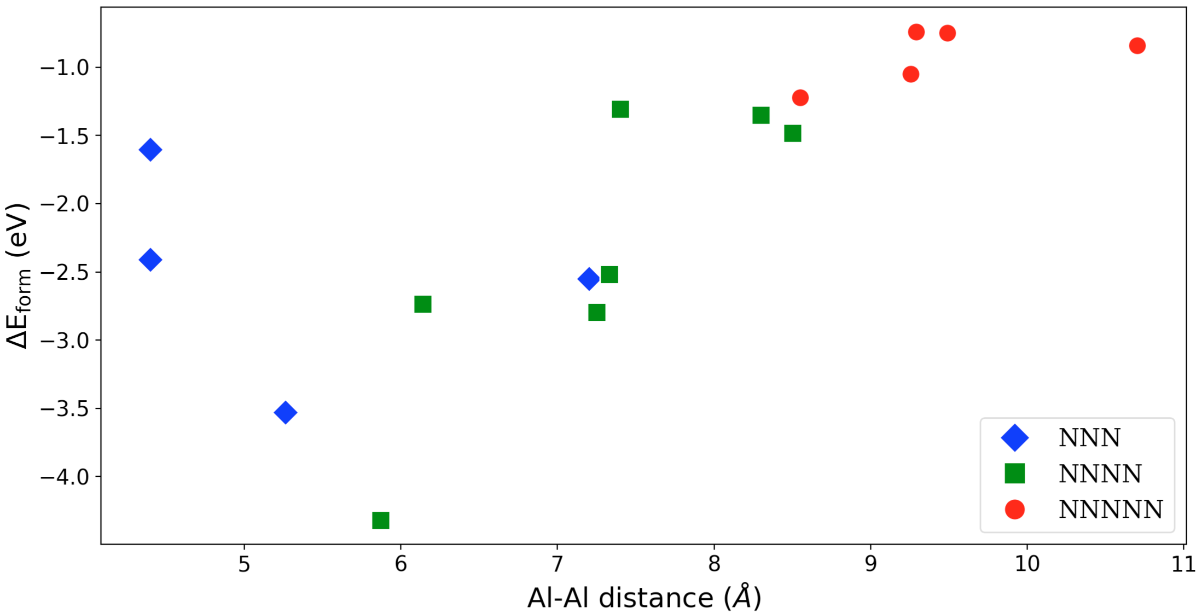

Supplementary Figure 2: Pd^{+2}-exchanged-CHA formation energy as a function of Al-Al distance. Color coding refers to the type of Al pairs.

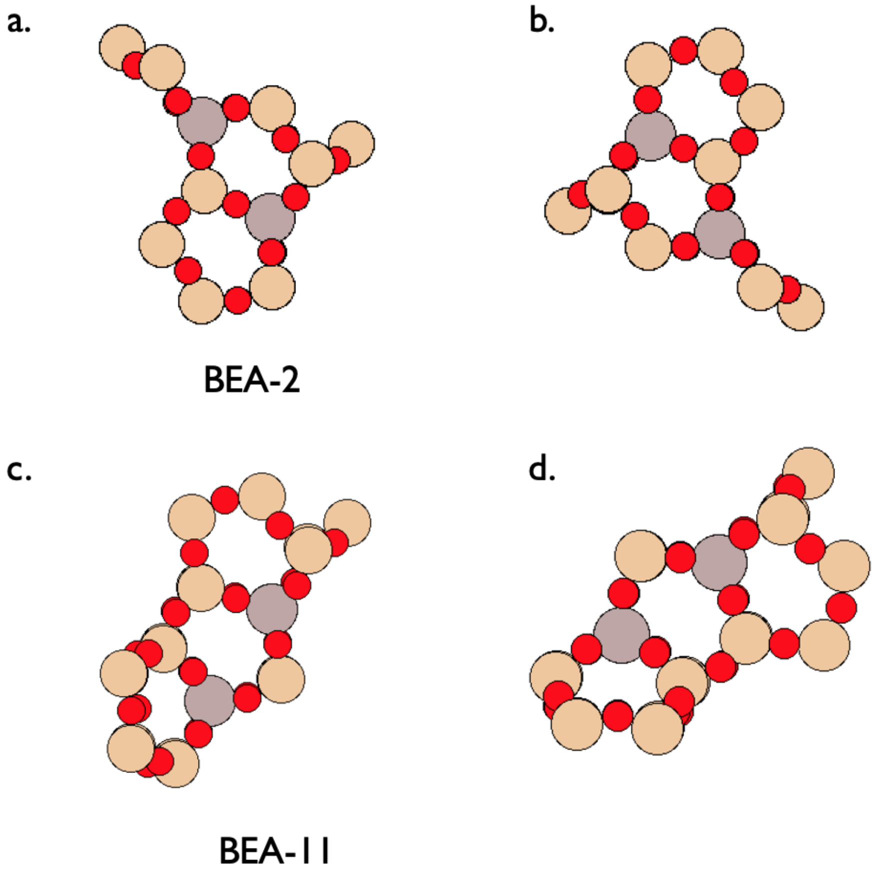

Supplementary Figure 3: Images of the framework QM atoms in four BEA structures. BEA-2 structure is shown in a. alongside a similar structure in b. BEA-11 is shown in c. alongside a similar structure in d. The structures similar to BEA-2 and BEA-11 (b. and d., respectively) are not exactly identical, as calculated by the nuclear repulsion energy, but share the same connectivity to surrounding Si and O atoms (types of MR). Color coding: red=oxygen, beige=silicon, grey=aluminum.

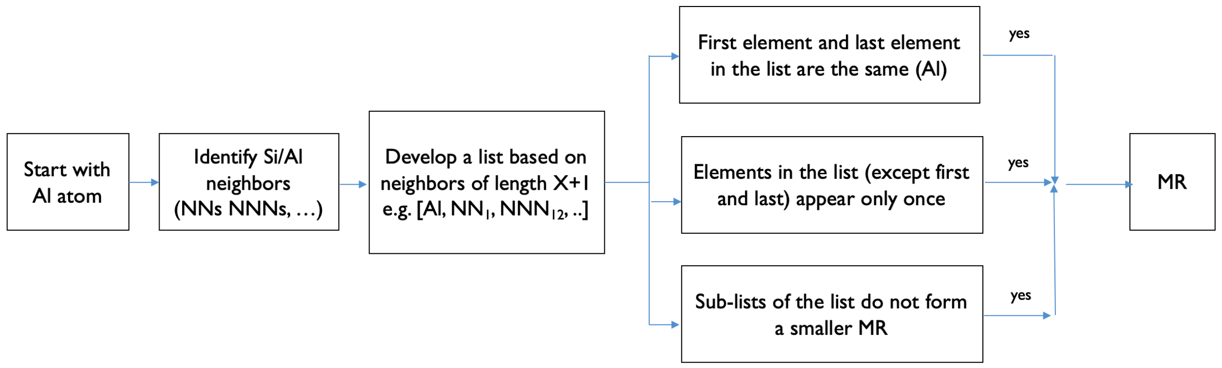

Supplementary Figure 4: Flow diagram for determining if Al atom is in an X MR (where X refers to the length of the MR). First, starting with an Al atom, Si/Al neighbors (NN, NNN, NNNN) are identified similar to the procedure described in Figure 2. This is done up to the X+1 neighbor. Based on the neighbors, lists are developed (an example of a list is [Al, NN_1, NNN_{11}, ..], where NN_1 refers to the first NN of the starting Al and NNN_{11} is the first NN to NN_1). For each list, the following checks are made: first and last element of the list are the same (the starting Al atom), no element in the list (with the exception of the starting Al atom) appears twice in the list, and no sub-list of the list form a MR smaller than X. If a list passes those checks, then Al is part of the X MR. Note that sub-lists are created in the same manner as a list. They start with the same initial Al atom, and include elements of length 4 to X. If a sub-list satisfies the first two checks (first and last elements are the same, and no element appears twice except the first and last ones), then a sub-list forms an X MR. Thus, the considered list does not form a MR.

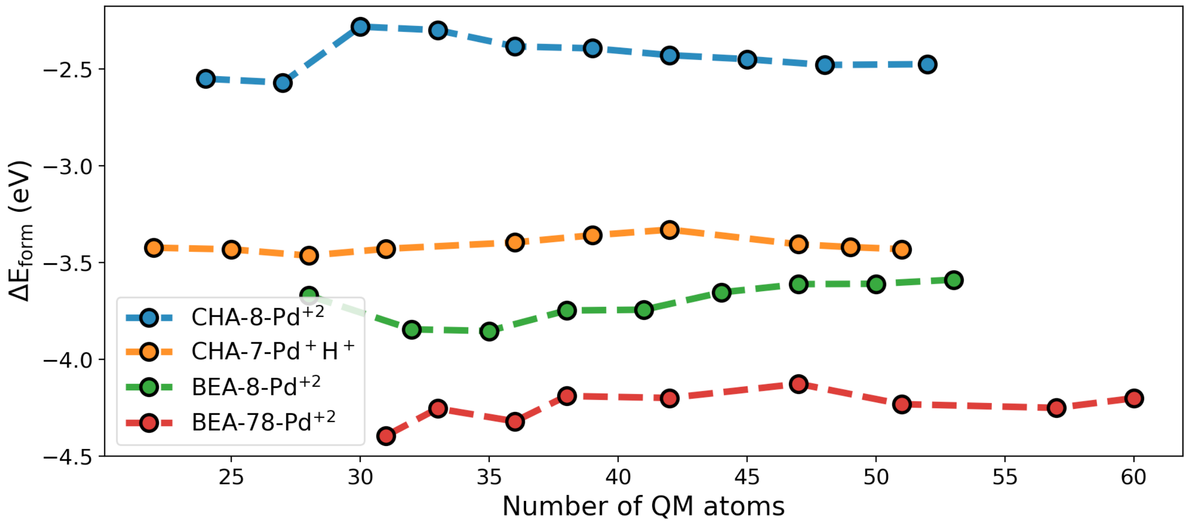

Supplementary Figure 5: Impact of the number of atoms selected in the QM region on the convergence of the QM/MM calculations (ΔE_{form} is defined in equation 2). Calculations were done using ωB97X-D functional.

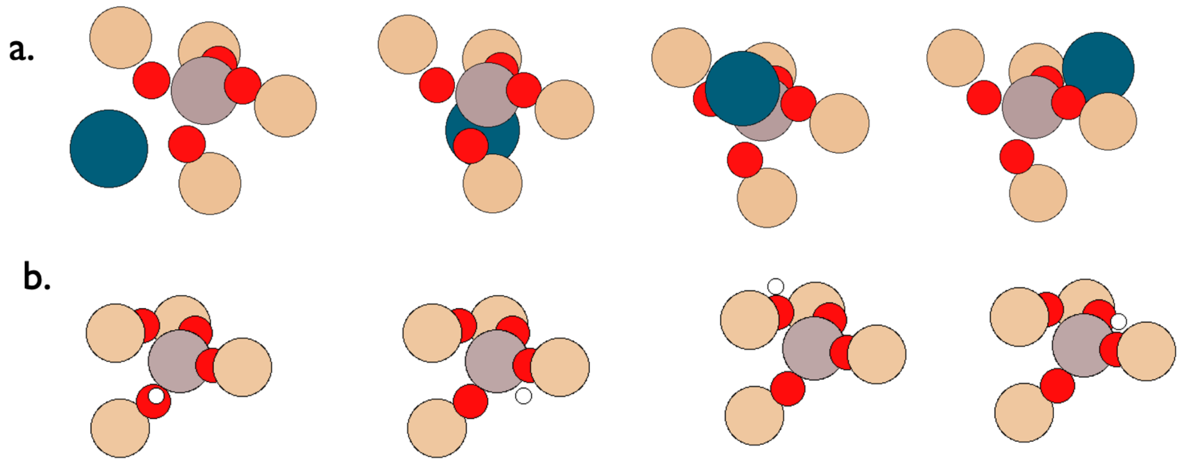

Supplementary Figure 6: Example of a. Pd cation and b. proton placement near the four oxygens neighboring Al atom. For clarity, only Si in NN position and O neighboring Al are shown. The position of cation/proton is determined by finding the middle distance between neighboring oxygen, and then adding a displacement from the Al atom (usually 1-1.5 A). Color coding: red=oxygen, beige=silicon, grey=aluminum, white=hydrogen, blue=palladium.

Supplementary Table 1: Comparison of the stability order (from most stable to 5th most stable) on 3 different structures (BEA-9, BEA-63 and BEA-76) at two levels of theory (GGA and hGGA). The adsorbate is Pd^+H^+. For simplicity, only the 5 most stable structures are shown out of the 32. The two results do not always yield the same order, however, the most stable structure at the hGGA level always appears among the 5 most stable structures at the GGA level. This means following the approach in Figure 1 yields the same results as doing all the calculations using ωB97X-D.

Stability	Theory Level	BEA-9	BEA-63	BEA-76
1st	GGA	BEA-9-Pd^+H^+-3	BEA-63-Pd^+H^+-24	BEA-76-Pd^+H^+-26
	hGGA	BEA-9-Pd^+H^+-12	BEA-63-Pd^+H^+-24	BEA-76-Pd^+H^+-25
2nd	GGA	BEA-9-Pd^+H^+-11	BEA-63-Pd^+H^+-4	BEA-76-Pd^+H^+-25
	hGGA	BEA-9-Pd^+H^+-11	BEA-63-Pd^+H^+-23	BEA-76-Pd^+H^+-26
3rd	GGA	BEA-9-Pd^+H^+-12	BEA-63-Pd^+H^+-3	BEA-76-Pd^+H^+-5
	hGGA	BEA-9-Pd^+H^+-3	BEA-63-Pd^+H^+-1	BEA-76-Pd^+H^+-5
4th	GGA	BEA-9-Pd^+H^+-24	BEA-63-Pd^+H^+-27	BEA-76-Pd^+H^+-24
	hGGA	BEA-9-Pd^+H^+-24	BEA-63-Pd^+H^+-27	BEA-76-Pd^+H^+-24
5th	GGA	BEA-9-Pd^+H^+-27	BEA-63-Pd^+H^+-1	BEA-76-Pd^+H^+-16
	hGGA	BEA-9-Pd^+H^+-27	BEA-63-Pd^+H^+-4	BEA-76-Pd^+H^+-16

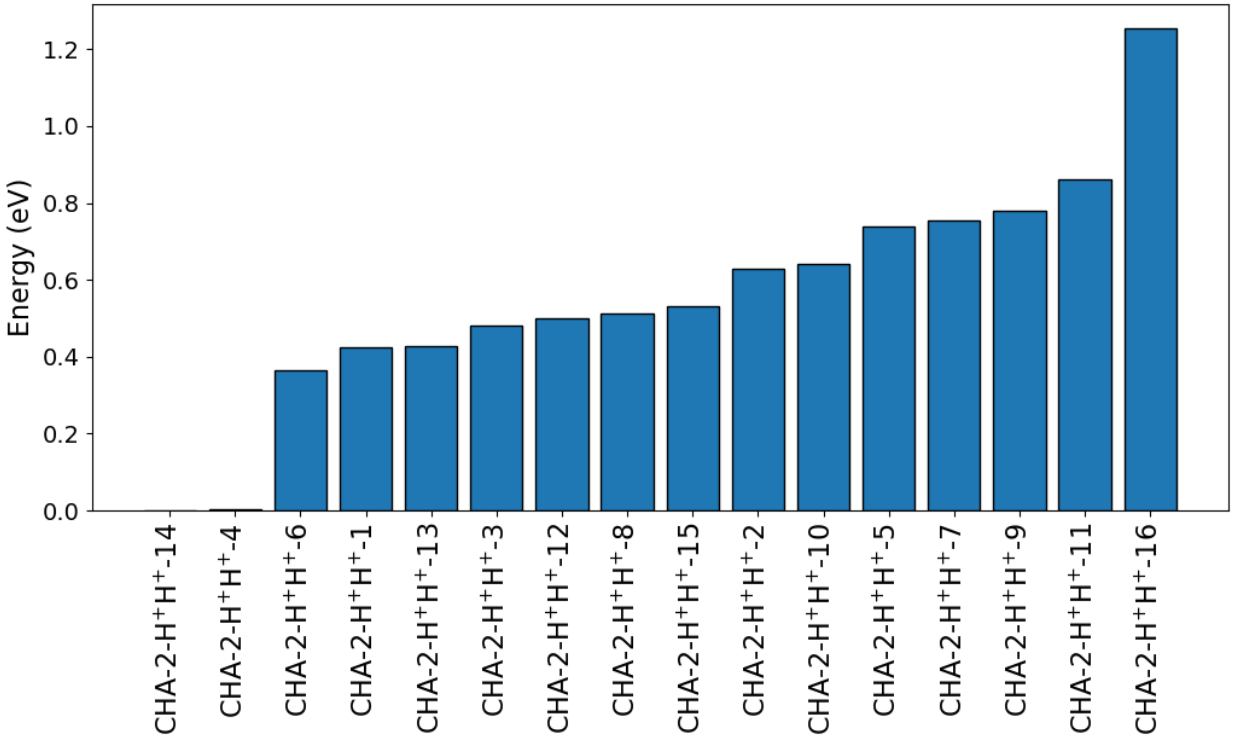

Supplementary Figure 7: CHA-2 optimum proton locations search results. Each data represents the optimum energy based on a different initial position of the protons. The x-axis refers to the index of the structure in the database in the SI and the y-axis is the energy relative to the most stable structure (CHA-2-H^+H^+-14). Calculations were done using the ωB97X-D functional.

Supplementary Table 2: Difference in NO adsorption energy (ΔE_{NO}) on Pd^+H^+ in CHA when the two electrons (from Pd^+ and NO) are paired and the two electrons are unpaired. Paired electrons are used as the reference (0 eV)

Structure Name	Paired Electrons	Two Unpaired Electrons
CHA-5	0	0.89
CHA-8	0	0.82
CHA-7	0	1.53

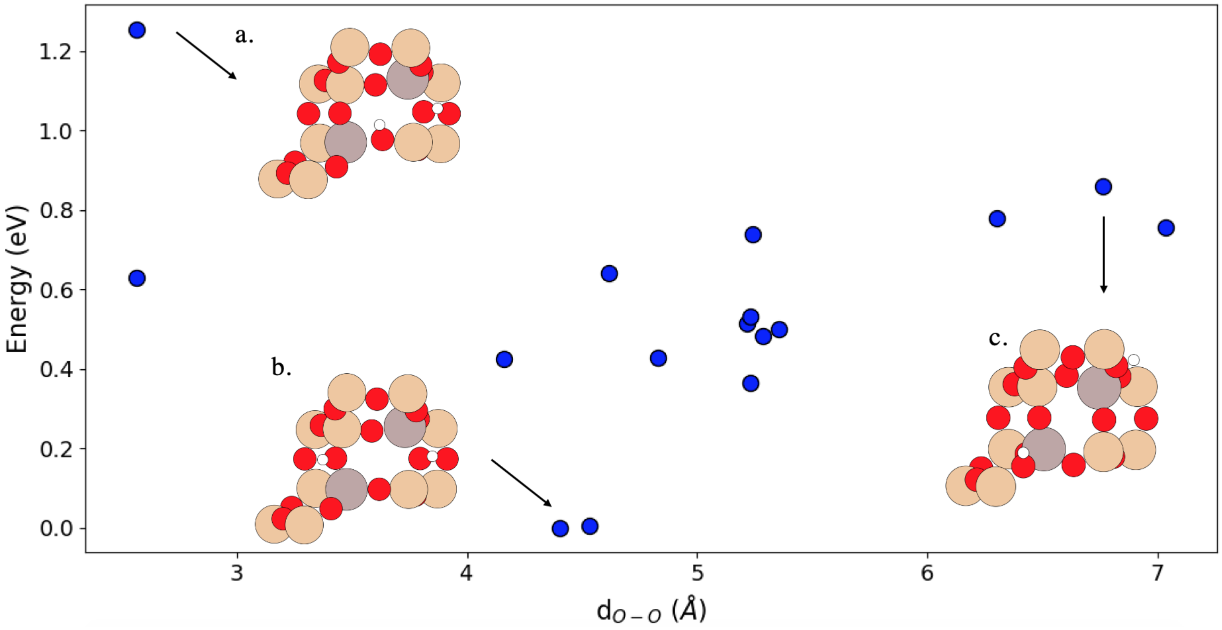

Supplementary Figure 8: CHA-2 optimum proton locations search results. Each data represents a different initial position of the protons. The x-axis refers to the O-O distance (where the oxygen is the atom H adsorbs on) in the optimized structure and the y-axis is the energy of the optimized structure relative to the most stable structure (CHA-2-H^+H^+-14). Insert images of select structures are shown (a. CHA-2-H^+H^+-2 b. CHA-2-H^+H^+-14 c. CHA-2-H^+H^+-7). Structures of all the other data points are available in the ESI.

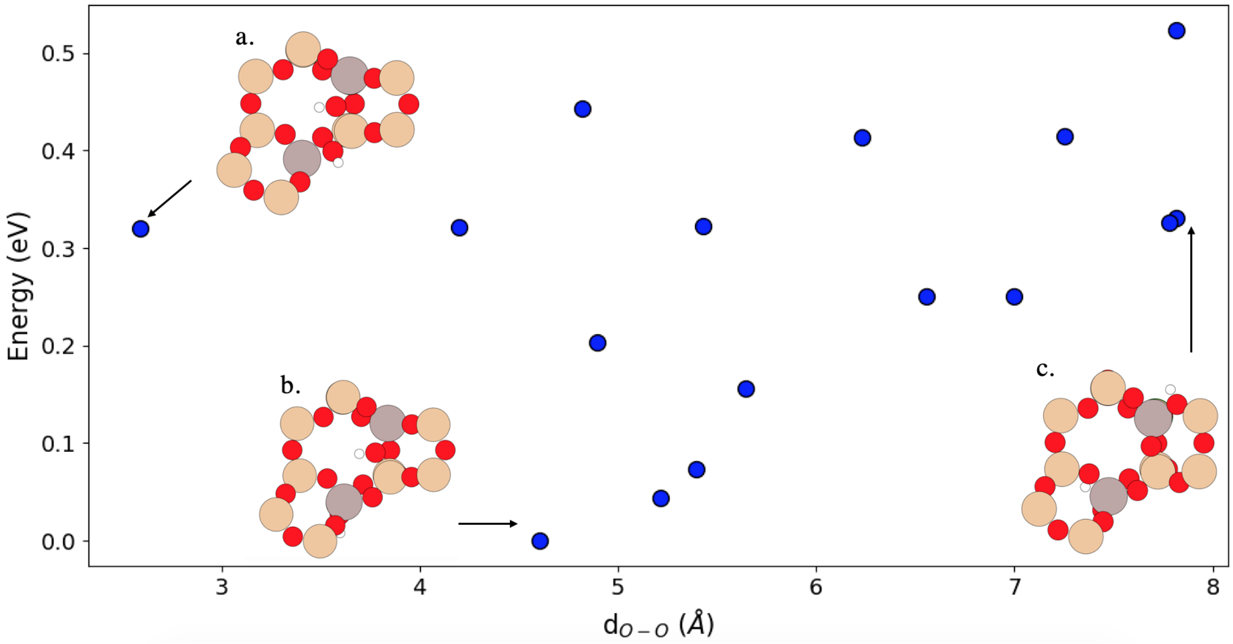

Supplementary Figure 9: CHA-3 optimum proton locations search results. Each data represents a different initial position of the protons. The x-axis refers to the O-O distance (where the oxygen is the atom H adsorbs on) in the optimized structure and the y-axis is the energy of the optimized structure relative to the most stable structure (CHA-3-H^+H^+-3). Insert images of select structures are shown (a. CHA-3-H^+H^+-1 b. CHA-3-H^+H^+-3 c. CHA-3-H^+H^+-11). Structures of all the other data points are available in the ESI.

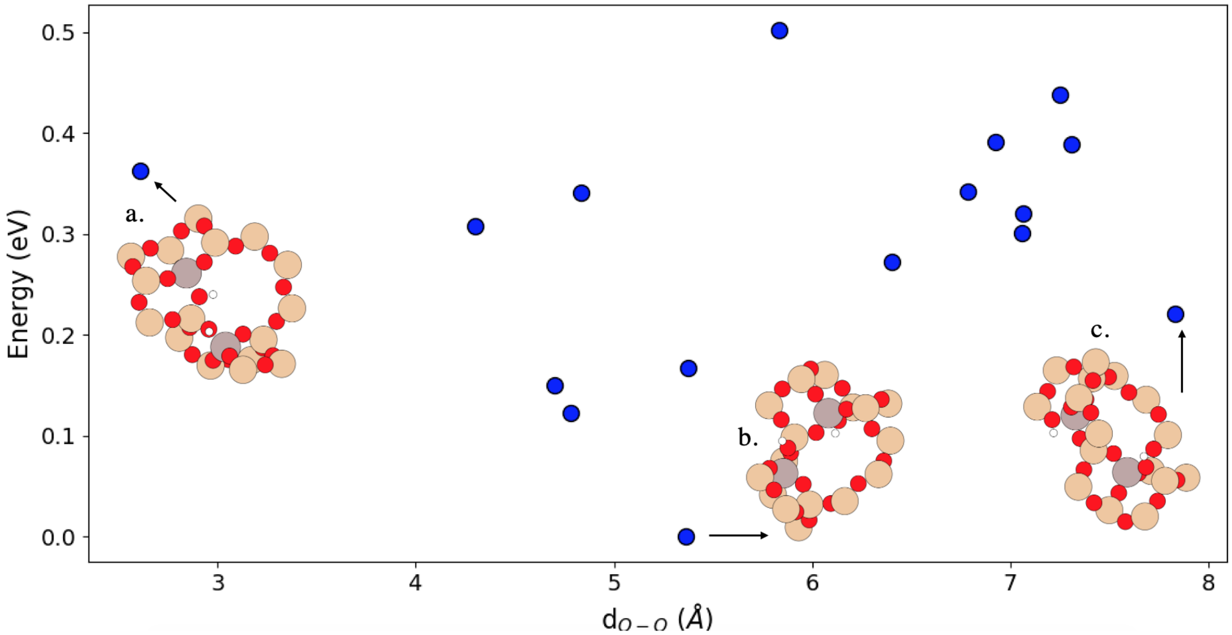

Supplementary Figure 10: CHA-5 optimum proton locations search results. Each data represents a different initial position of the protons. The x-axis refers to the O-O distance (where the oxygen is the atom H adsorbs on) in the optimized structure and the y-axis is the energy of the optimized structure relative to the most stable structure (CHA-5-H^+H^+-3). Insert images of select structures are shown (a. CHA-5-H^+H^+-7 b. CHA-5-H^+H^+-3 c. CHA-5-H^+H^+-4). Structures of all the other data points are available in the ESI.

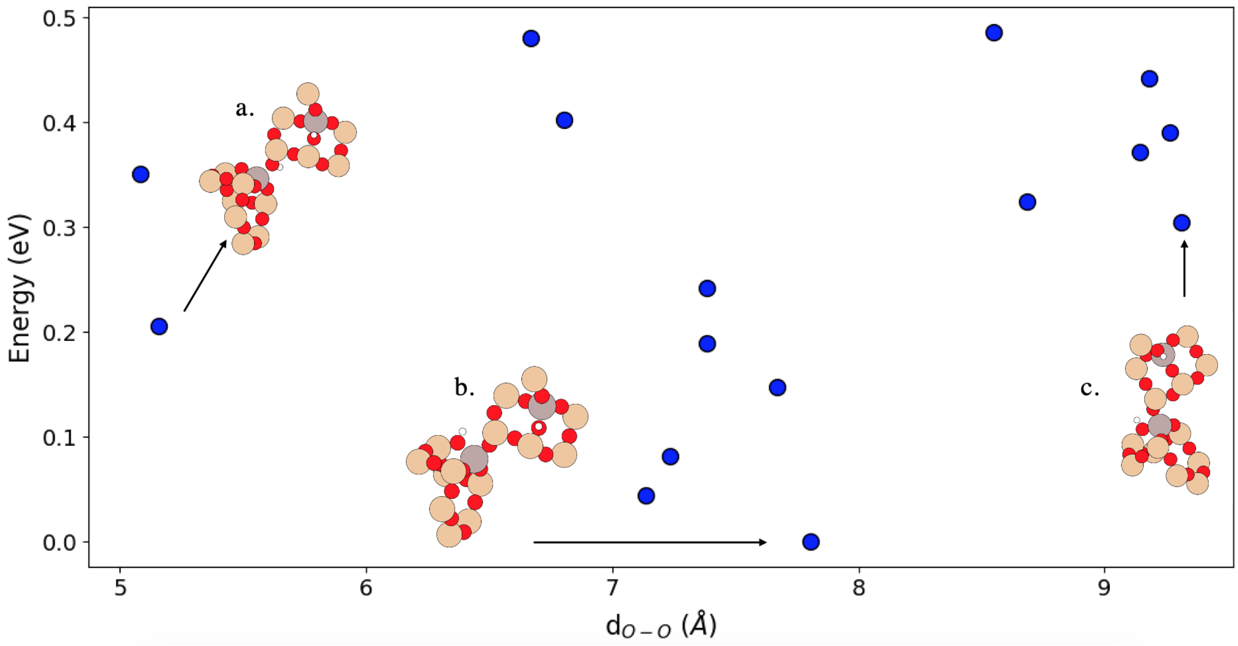

Supplementary Figure 11: CHA-6 optimum proton locations search results. Each data represents a different initial position of the protons. The x-axis refers to the O-O distance (where the oxygen is the atom H adsorbs on) in the optimized structure and the y-axis is the energy of the optimized structure relative to the most stable structure (CHA-6-H^+H^+-4). Insert images of select structures are shown (a. CHA-6-H^+H^+-2 b. CHA-6-H^+H^+-4 c. CHA-6-H^+H^+-8). Structures of all the other data points are available in the ESI.

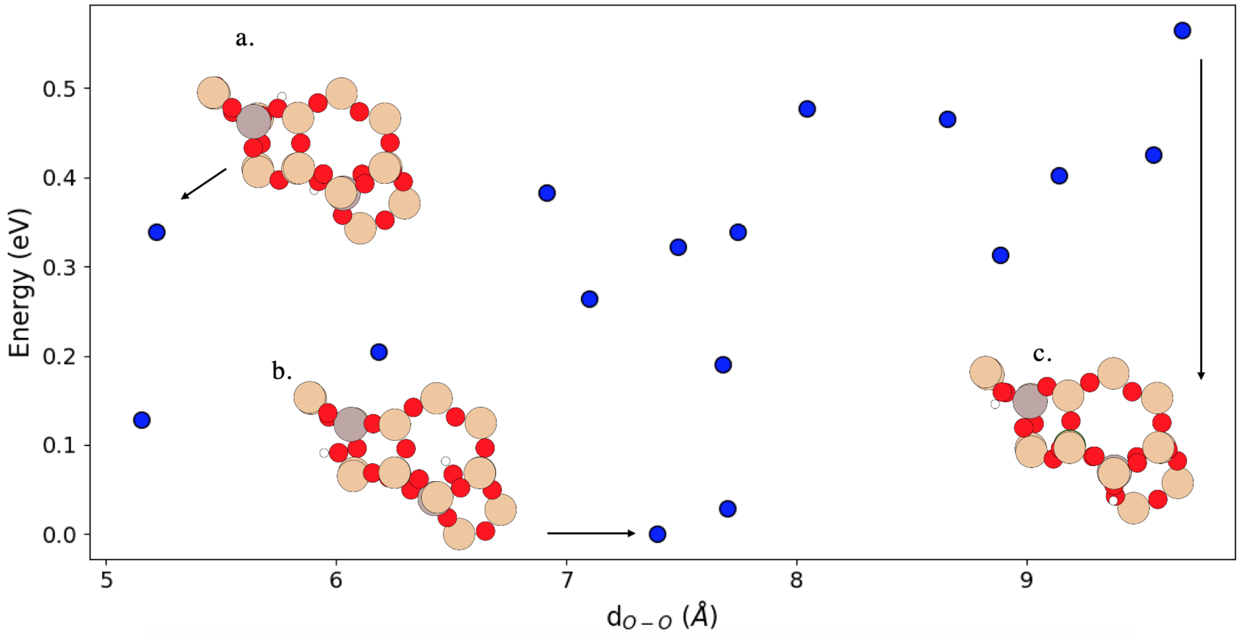

Supplementary Figure 12: CHA-8 optimum proton locations search results. Each data represents a different initial position of the proton. The x-axis refers to the O-O distance (where the oxygen is the atom H adsorbs on) in the optimized structure and the y-axis is the energy of the optimized structure relative to the most stable structure (CHA-8-H^+H^+-1). Insert images of select structures are shown (a. CHA-8-H^+H^+-10 b. CHA-8-H^+H^+-1 c. CHA-8-H^+H^+-7). Structures of all the other data points are available in the ESI.

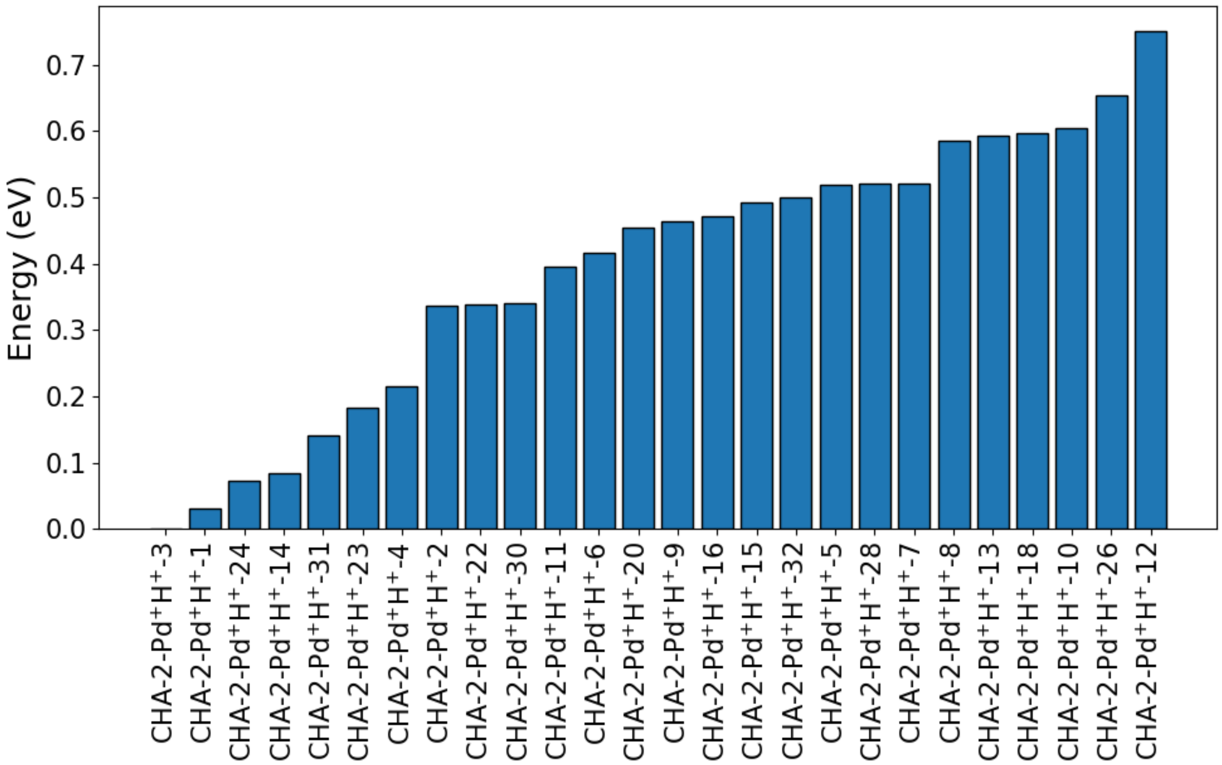

Supplementary Figure 13: CHA-2 optimum Pd^+H^+ locations search results. Each data represents a different initial position of Pd^+H^+. The x-axis refers to the index of the structure in the database and the y-axis is the energy relative to the most stable structure (CHA-2-Pd^+H^+-3). Calculations were done using the ωB97X-D functional. Only 26 of the 32 possibilities are shown due to lack of convergence for 6 structures. These structures failed to converge due to unfavorable initial conditions, as stated in the main manuscript.

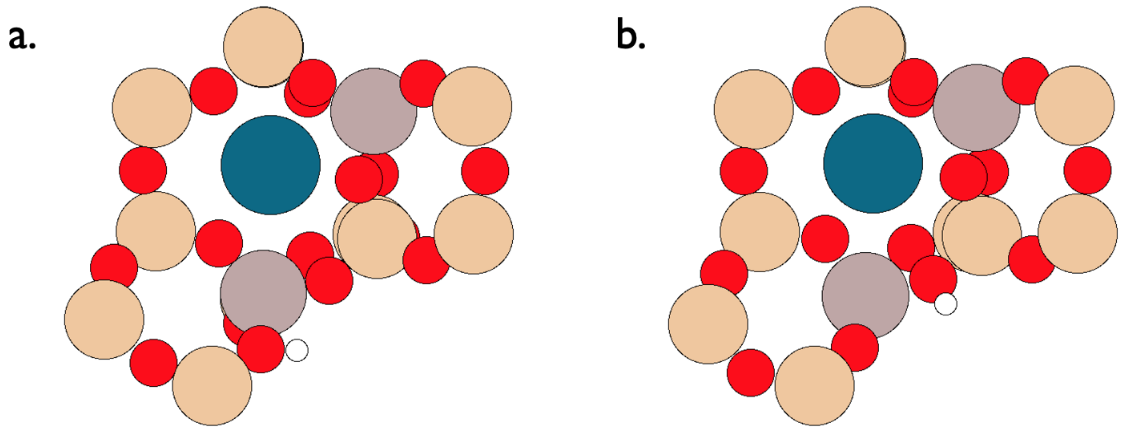

Supplementary Figure 14: QM images of the optimized geometry of a. CHA-3-$Pd^{+}H^{+}$-21 and b. CHA-3-$Pd^{+}H^{+}$-17. Despite only a change in the proton position, there is >0.4 eV in energy difference between the two optimized geometries. The color coding is the same as in Figure 5.

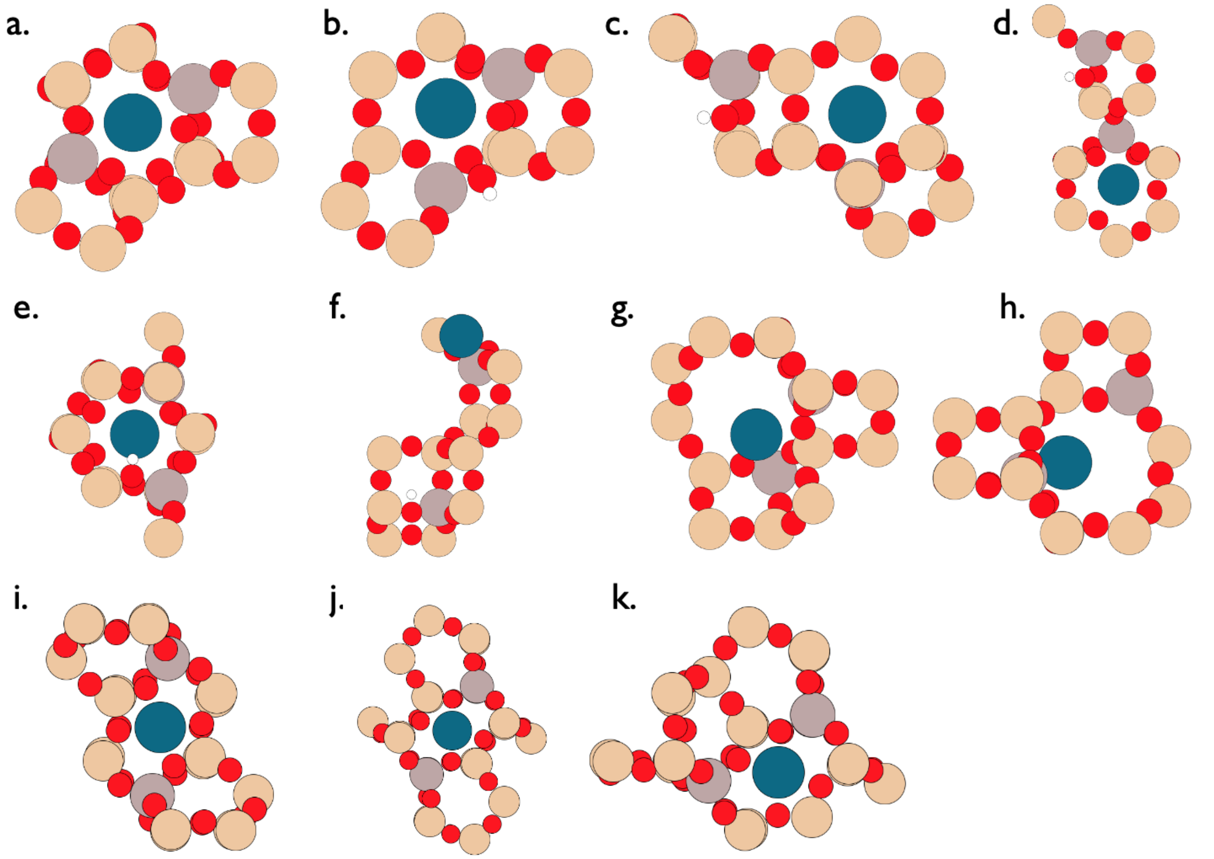

Supplementary Figure 15: QM region images of the optimized geometry of a. $Pd^{+}H^{+}$ on CHA-7 b. $Pd^{+}H^{+}$ on CHA-3 c. $Pd^{+}H^{+}$ on CHA-8 d. $Pd^{+}H^{+}$ on CHA-6 e. $Pd^{+}H^{+}$ on CHA-9 f. $Pd^{+}H^{+}$ on CHA-12 g. Pd^{+2} on CHA-5 and h. Pd^{+2} on CHA-10. i. Pd^{+2} on BEA-95 j. Pd^{+2} on BEA-55 k. Pd^{+2} on BEA-48. The color coding is the same as in Figure 5.

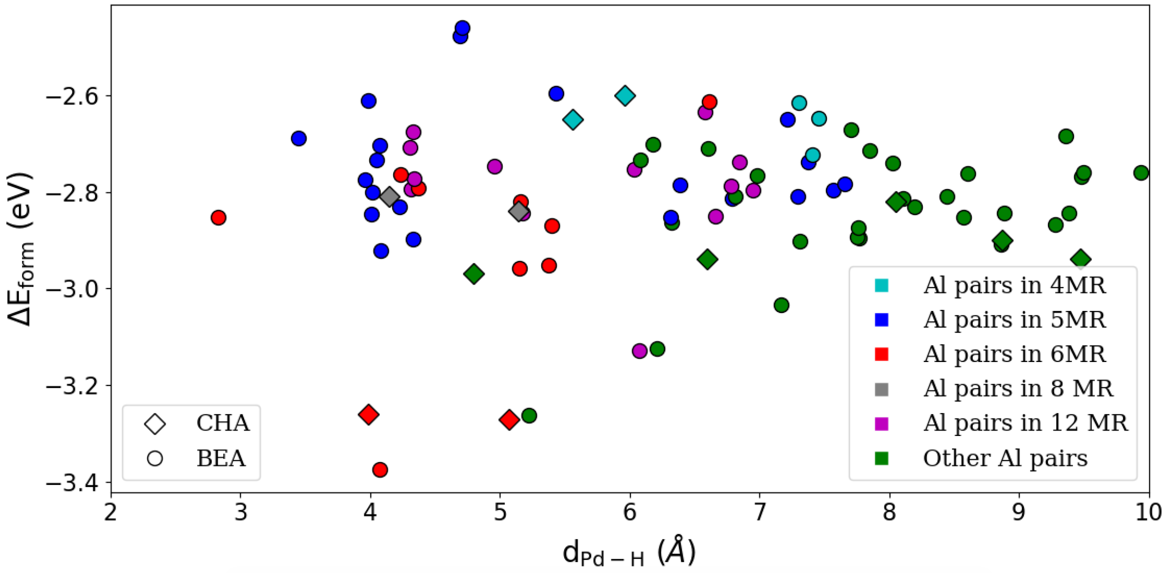

Supplementary Figure 16: ΔE_{form} as a function of the distance between Pd and H in $Pd^{+}H^{+}$ optimized geometry on CHA ($\Diamond$) and BEA ($\circ$).

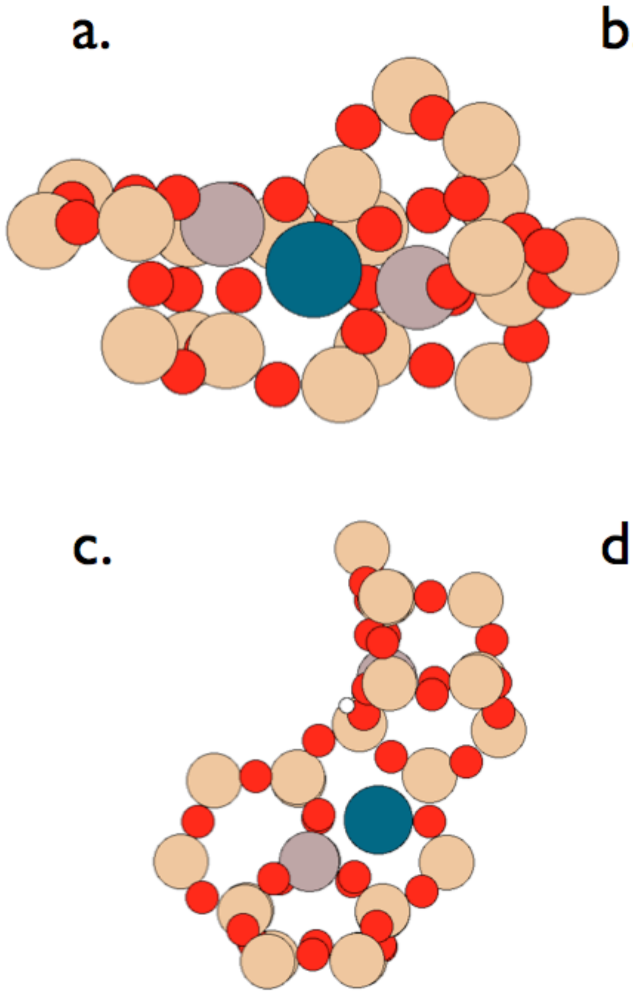

Supplementary Figure 17: QM region images of optimized geometry of a. Pd^{+2} on BEA-8 b. Pd^{+2} on BEA-66 c. $Pd^{+}H^{+}$ on BEA-49 d. $Pd^{+}H^{+}$ on BEA-66. The color coding is the same as in Figure 5.

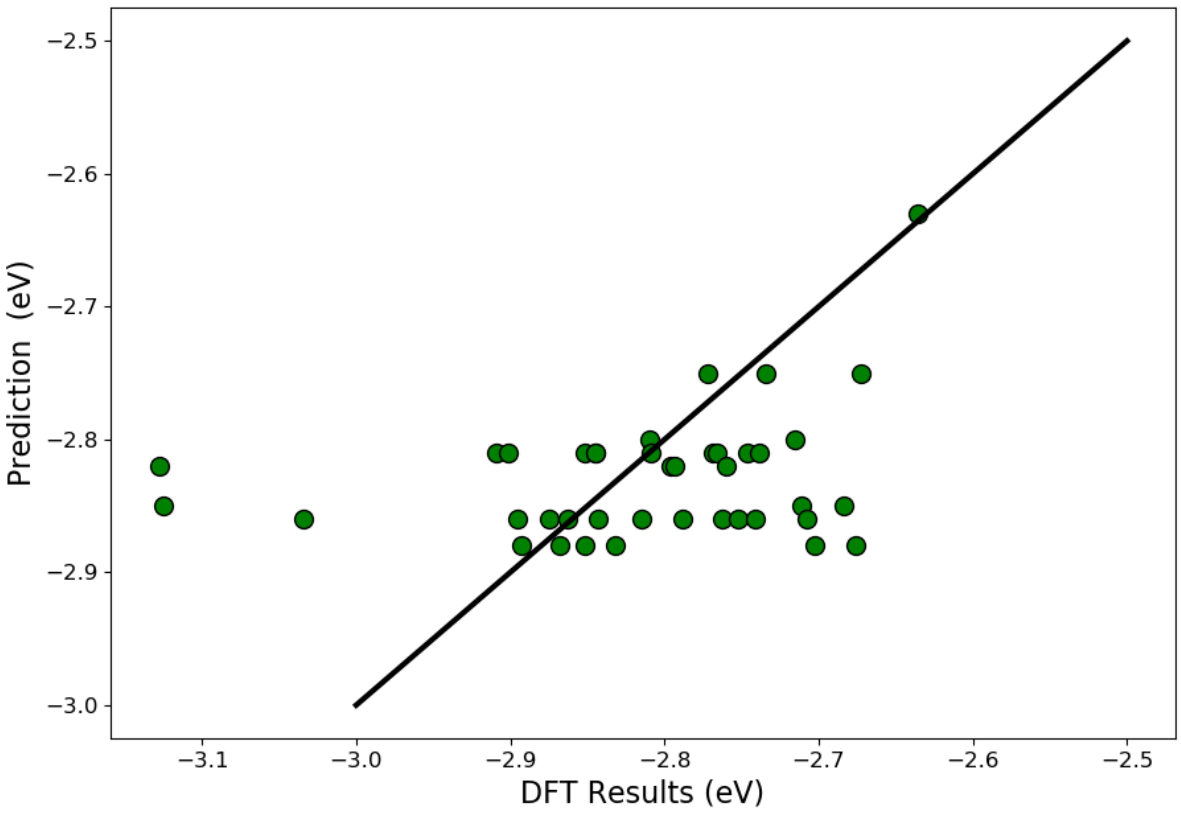

Supplementary Figure 18: Parity plot of the calculated DFT energy on Al pairs (that do not share a MR) and the predicted energy based on the respective isolated Al site (the most stable of the two) (R-squared=0.086). All calculations were done on BEA with $Pd^{+}H^{+}$ as the cation.

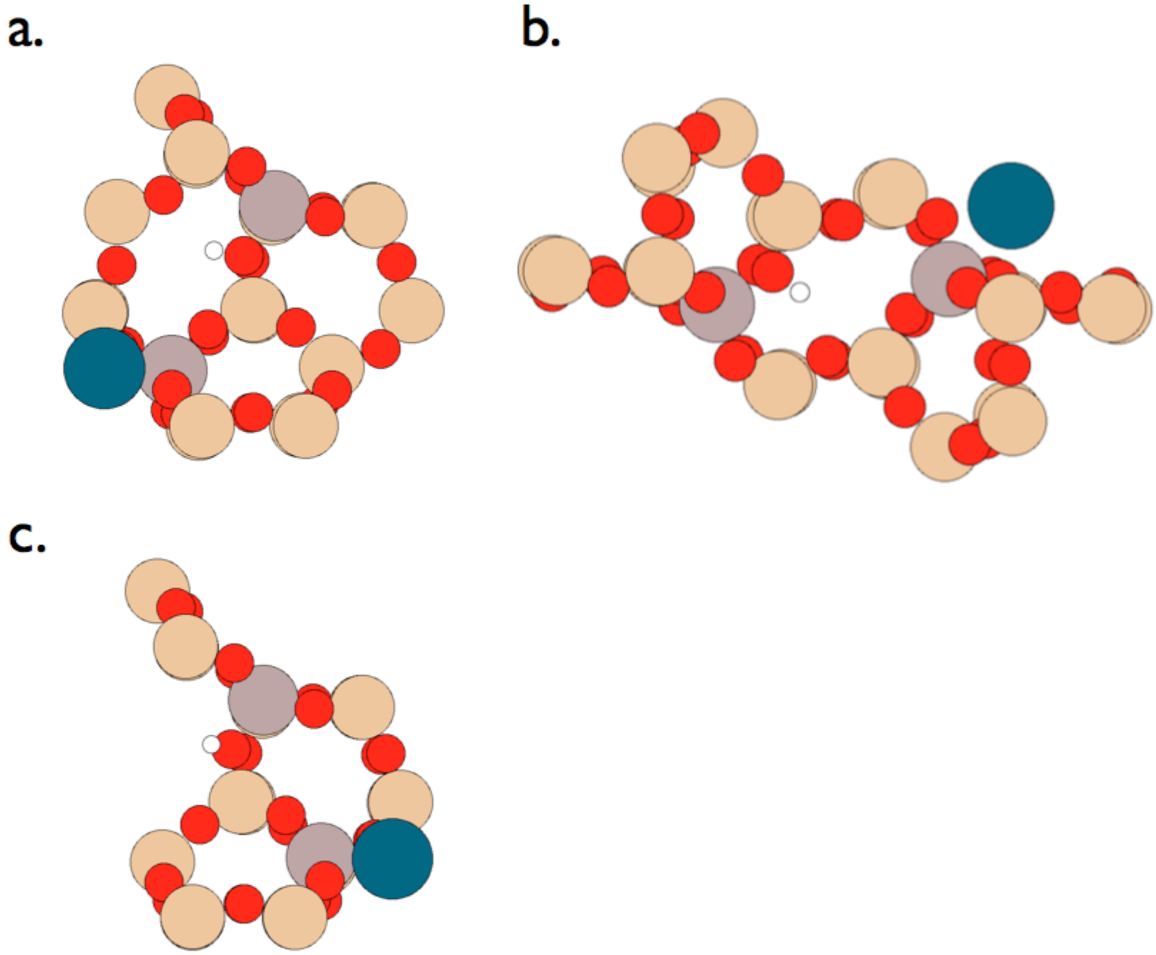

Supplementary Figure 19: Image of the QM region on optimized BEA calculations a. BEA-62 ($Pd^{+}H^{+}$) b. BEA-93 ($Pd^{+}H^{+}$) c. BEA-63 ($Pd^{+}H^{+}$). The color coding is the same as in Figure 5.

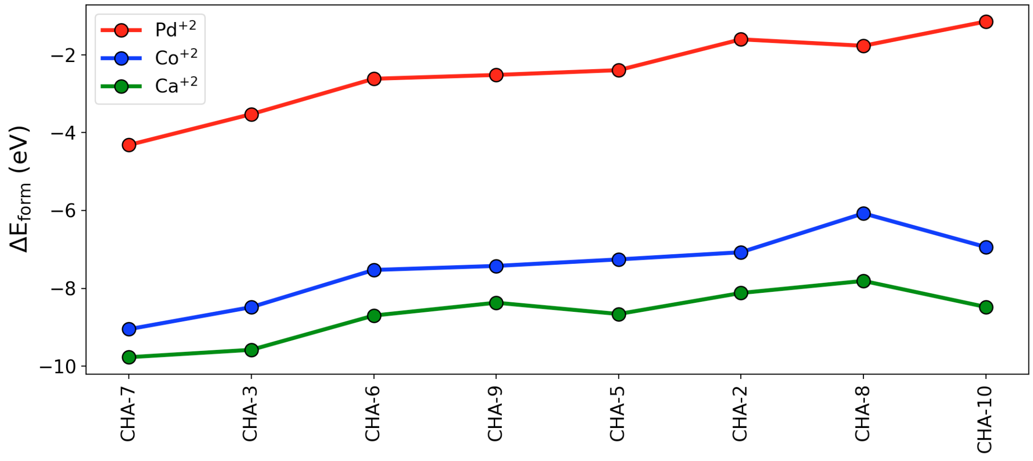

Supplementary Figure 20: Comparison between favorable Al pair configurations for Pd^{+2}, Co^{+2}, and Ca^{+2} exchanged-CHA.

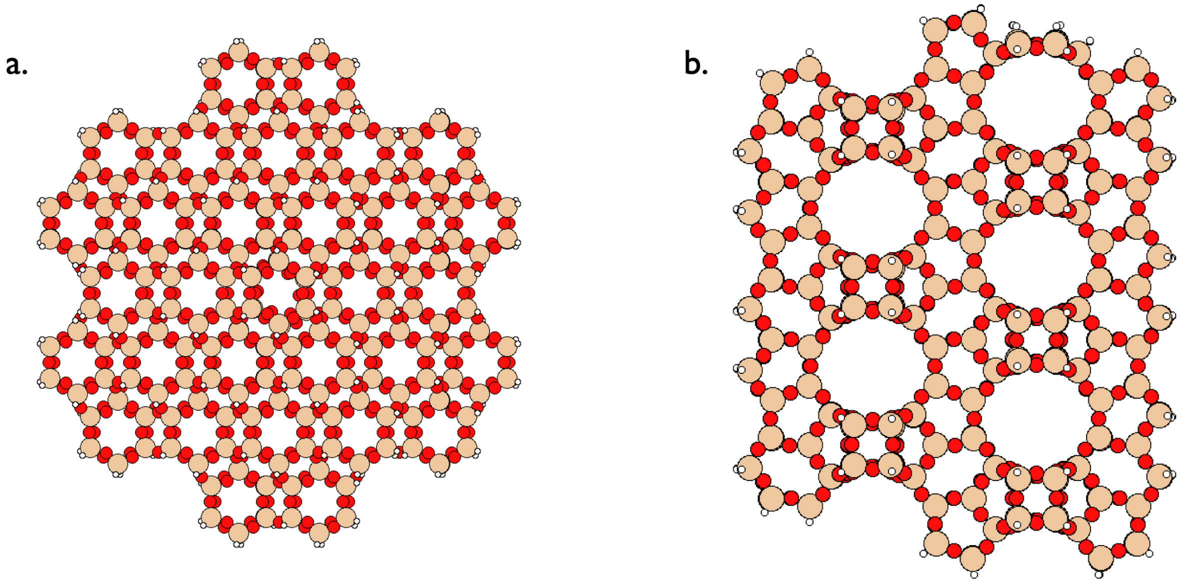

Supplementary Figure 21: Cluster models of a. T696 CHA and b. T810 BEA. The color coding is the same as in Figure 5.

.
